# Correction: Yamamoto et al. Trends in Open vs. Endoscopic Carpal Tunnel Release: A Comprehensive Survey in Japan. *J. Clin. Med.* 2022, *11*, 4966

**DOI:** 10.3390/jcm12062223

**Published:** 2023-03-13

**Authors:** Michiro Yamamoto, James Curley, Hitoshi Hirata

**Affiliations:** Department of Hand Surgery, Nagoya University Graduate School of Medicine, 65 Tsurumai-cho, Showa-ku, Nagoya 466-8550, Japan

In the original publication [1], there was a mistake in Figure 4 as published. 

The authors used the wrong diagram in Figure 4. The wrong diagram in Figure 4 is below.


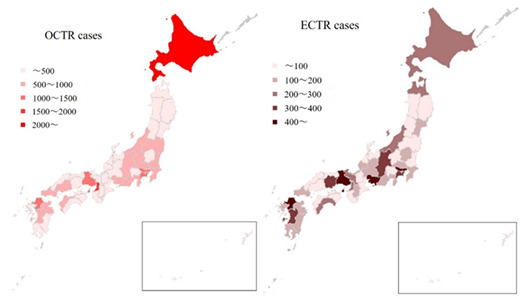


The corrected Figure 4 is below.


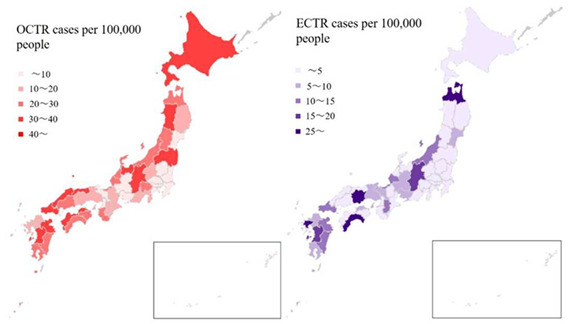


In addition, “(a)” at the end of the first sentence from the legend of Figure 3 was deleted.

The authors state that the scientific conclusions are unaffected. This correction was approved by the Academic Editor. The original publication has also been updated.
