# Supplementary material for: Trends in Open vs. Endoscopic Carpal Tunnel Release: A Comprehensive Survey in Japan
Source: J Clin Med. 2022 Aug 24;11(17):4966. doi: 10.3390/jcm11174966 (PMC9456605; doi:10.3390/jcm11174966)
Supplement: Supplementary file 1 [file jcm-11-04966-s001.zip › jcm-1854885-supplementary.pdf]

Table S1. Prefectural data.

Prefecture	Hand surgery specialists	Prefectural residents (thousands)	Hand surgery specialists per 100,000 residents	OCTR SIR (95% CI)	ECTR SIR (95% CI)
Hokkaido	58	5250	1.11	1.55 (1.51–1.6)	0.66 (0.41–0.91)
Aomori	10	1246	0.8	1.22 (0.99–1.44)	3.40 (3.02–3.78)
Iwate	8	1227	0.65	0.99 (0.82–1.17)	0.26 (0.01–0.51)
Miyagi	9	2306	0.39	0.68 (0.58–0.78)	1.00 (0.35–1.66)
Akita	2	966	0.21	1.56 (1.47–1.66)	0.05 (0.0–0.14)
Yamagata	14	1078	1.3	1.28 (1.22–1.34)	0.70 (0.35–1.04)
Fukushima	12	1846	0.65	1.54 (1.39–1.69)	0.28 (0.16–0.39)
Ibaragi	12	2860	0.42	0.78 (0.68–0.89)	0.29 (0.20–0.37)
Tochigi	15	1934	0.78	0.8 (0.72–0.87)	1.12 (0.86–1.38)
Gunma	17	1942	0.88	1.12 (1.01–1.24)	0
Saitama	33	7350	0.45	0.39 (0.32–0.46)	0.36 (0.27–0.44)
Chiba	42	6259	0.67	0.67 (0.59–0.75)	0.40 (0.28–0.52)
Tokyo	150	13921	1.08	0.5 (0.43–0.58)	0.73 (0.63–0.83)
Kanagawa	57	9198	0.62	0.58 (0.53–0.63)	0.63 (0.51–0.75)
Niigata	30	2223	1.35	1.23 (1.14–1.32)	1.78 (0.68–2.88)
Toyama	5	1044	0.48	1.57 (1.39–1.76)	0.71 (0.24–1.18)
Ishikawa	9	1138	0.79	1.18 (1–1.37)	0.13 (0.0–0.32)
Fukui	5	768	0.65	1.21 (1.09–1.34)	2.16 (1.51–2.81)
Yamanashi	7	811	0.86	1.14 (1.06–1.22)	0
Nagano	22	2049	1.07	1.73 (1.63–1.83)	2.63 (2.38–2.89)
Gifu	10	1987	0.5	0.65 (0.61–0.7)	0.97 (0.58–1.36)
Shizuoka	21	3644	0.58	1.05 (0.98–1.13)	0.81 (0.74–0.88)
Aichi	62	7552	0.82	0.36 (0.33–0.39)	1.28 (0.96–1.59)
Mie	10	1781	0.56	0.93 (0.89–0.97)	0.72 (0.32–1.12)
Shiga	7	1414	0.5	0.86 (0.78–0.93)	0.25 (0.04–0.46)
Kyoto	27	2583	1.05	0.78 (0.68–0.89)	1.41 (1.20–1.63)
Osaka	92	8809	1.04	0.76 (0.67–0.85)	0.48 (0.30–0.66)
Hyogo	38	5466	0.7	0.82 (0.75–0.89)	1.28 (0.94–1.63)
Nara	16	1330	1.2	1.05 (1.01–1.09)	2.04 (1.58–2.50)
Wakayama	11	925	1.19	1.33 (1.17–1.5)	0.33 (0.15–0.51)

Tottori	2	556	0.36	1.68 (1.44–1.92)	0.79 (0.09–1.48)
Shimane	5	674	0.74	1.94 (1.75–2.14)	0
Okayama	22	1890	1.16	0.9 (0.84–0.97)	3.45 (1.99–4.90)
Hiroshima	26	2804	0.93	1.3 (1.25–1.35)	0.58 (0.45–0.71)
Yamaguchi	17	1358	1.25	1.55 (1.48–1.62)	1.70 (1.49–1.91)
Tokushima	4	728	0.55	1.17 (0.99–1.34)	0
Kagawa	12	956	1.26	1.41 (1.33–1.49)	1.02 (0.93–1.22)
Ehime	6	1339	0.45	1.52 (1.44–1.6)	0.76 (0.25–1.27)
Kochi	7	698	1	1.25 (1.1–1.41)	4.45 (3.79–5.11)
Fukuoka	48	5104	0.94	1.07 (0.96–1.19)	1.39 (1.00–1.78)
Saga	9	815	1.1	1.1 (0.91–1.3)	3.92 (2.72–5.12)
Nagasaki	9	1327	0.68	0.86 (0.8–0.92)	0.24 (0.06–0.41)
Kumamoto	10	1748	0.57	2.22 (2.1–2.34)	3.08 (2.23–3.92)
Oita	11	1135	0.97	1.83 (1.63–2.03)	2.07 (0.96–3.17)
Miyazaki	4	1073	0.37	1.28 (1.14–1.41)	1.93 (1.67–2.19)
Kagoshima	5	1602	0.31	1.16 (1.02–1.3)	1.26 (1.16–1.37)
Okinawa	11	1453	0.76	0.86 (0.77–0.95)	0.03 (0.0–0.11)

OCTR: open carpal tunnel release; ECTR: endoscopic carpal tunnel release; SIR: standardized incidence ratio; CI: confidence interval
